# Asymmetrical Gene Flow in a Hybrid Zone of Hawaiian Schiedea (Caryophyllaceae) Species with Contrasting Mating Systems

**DOI:** 10.1371/journal.pone.0024845

**Published:** 2011-09-19

**Authors:** Lisa E. Wallace, Theresa M. Culley, Stephen G. Weller, Ann K. Sakai, Ashley Kuenzi, Tilottama Roy, Warren L. Wagner, Molly Nepokroeff

**Affiliations:** 1 Department of Biological Sciences, Mississippi State University, Mississippi State, Mississippi, United States of America; 2 Department of Biological Sciences, University of Cincinnati, Cincinnati, Ohio, United States of America; 3 Department of Ecology and Evolutionary Biology, University of California Irvine, Irvine, California, United States of America; 4 Department of Biological Sciences, The State University of New York at Buffalo, Buffalo, New York, United States of America; 5 Department of Botany, Smithsonian Institution, Washington, D.C, United States of America; 6 Department of Biology, University of South Dakota, Vermillion, South Dakota, United States of America; University of Texas Arlington, United States of America

## Abstract

Asymmetrical gene flow, which has frequently been documented in naturally occurring hybrid zones, can result from various genetic and demographic factors. Understanding these factors is important for determining the ecological conditions that permitted hybridization and the evolutionary potential inherent in hybrids. Here, we characterized morphological, nuclear, and chloroplast variation in a putative hybrid zone between *Schiedea menziesii* and *S. salicaria*, endemic Hawaiian species with contrasting breeding systems. *Schiedea menziesii* is hermaphroditic with moderate selfing; *S. salicaria* is gynodioecious and wind-pollinated, with partially selfing hermaphrodites and largely outcrossed females. We tested three hypotheses: 1) putative hybrids were derived from natural crosses between *S. menziesii* and *S. salicaria*, 2) gene flow via pollen is unidirectional from *S. salicaria* to *S. menziesii* and 3) in the hybrid zone, traits associated with wind pollination would be favored as a result of pollen-swamping by *S. salicaria*. *Schiedea menziesii* and *S. salicaria* have distinct morphologies and chloroplast genomes but are less differentiated at the nuclear loci. Hybrids are most similar to *S. menziesii* at chloroplast loci, exhibit nuclear allele frequencies in common with both parental species, and resemble *S. salicaria* in pollen production and pollen size, traits important to wind pollination. Additionally, unlike *S. menziesii*, the hybrid zone contains many females, suggesting that the nuclear gene responsible for male sterility in *S. salicaria* has been transferred to hybrid plants. Continued selection of nuclear genes in the hybrid zone may result in a population that resembles *S. salicaria*, but retains chloroplast lineage(s) of *S. menziesii*.

## Introduction

Analyses of hybrid zones have provided a wealth of information on factors that favor interspecific hybridization, the nature of pre- and post-zygotic barriers to hybridization, and the stability of hybrid zones [1]–[3]. These studies provide insights into the role of hybridization in contemporary natural populations as well as past hybridization events. The structure and stability of hybrid zones depend on the extent to which parental species are genetically and ecologically distinct, their dispersal abilities, and the fitness of hybrid offspring [4]. When hybrids have lower fitness compared to their parents, a tension zone is formed and repeated hybridization is needed to maintain the hybrid zone [5], [6]. In contrast, when hybrids have higher fitness than their parents, they are likely to increase in frequency, and may displace adjacent parental populations to expand their range [5], [6].

Contrasts between maternally-inherited chloroplast DNA and nuclear DNA inherited through both parents can provide confirmation of hybridization and evidence for the direction of gene flow [7]–[11]. Asymmetric introgession is much stronger in organelle genes than nuclear genes because the former experiences lower rates of gene flow due to uniparental inheritance. Selection is often invoked to explain asymmetric patterns of introgression [12], [13]. For example, selection for greater pollen production or a more effective means of dispersing pollen could result in preferential gene flow to a species with lower pollen production or a less effective means of pollen dispersal. Demographic factors may also play an important role in determining the direction of introgression as Currat *et al.* [3] found strong evidence of asymmetrical introgression in a wide variety of taxa where gene flow occurred from a local to an invading species.

When hybridization occurs between selfing and outcrossing species, a rapidly advancing hybrid zone favoring the outcrossing species is expected to develop. For example, when dioecious, wind-pollinated populations of *Mercurialis annua* (Euphorbiaceae) came into contact with monoecious, more highly selfing populations of *M. annua*, the monoecious populations were swamped by pollen from the dioecious wind-pollinated populations [14]. Rapid movement of the hybrid zone favoring the dioecious form of *M. annua* was thought to result from differences in pollen flow related to the differing breeding systems within this species. Variation in pollinator preference could have similar effects on the outcome of hybridization. Campbell *et al.* [15] described an ‘advancing wave’ dynamic for *Ipomopsis aggregata* and *I. tenuituba* (Polemoniaceae). In the hybrid zone between these two species, traits of *I. aggregata* were favored because hummingbirds preferentially visit *I. aggregata* and are much more common pollinators than the hawkmoths that favor *I. tenuituba*. In experimental arrays, *I. aggregata* received much more pollen from conspecifics and hybrids than *I. tenuituba* [16]. Similar array studies demonstrated that hybridization between species of *Castilleja* (Orobanchaceae) was also influenced by pollinator constancy, which was in turn determined by the frequency of hybrids, leading to the possibility of a self-reinforcing mechanism creating areas where hybrids might be very common [17]. Finally, in a hybrid zone between *Penstemon davidsonii* and *P. newberryi* (Plantaginaceae), gene flow from the high-elevation *P. davidsonii* to the lower elevation *P. newberryi* was mediated primarily through frequent visits by hummingbirds and other pollinators to both hybrids and *P. newberryi* [18]. Consequently, knowledge of pollinator behavior and the reproductive system are important for predicting the outcome of hybridization and the movement of current hybrid zones across the landscape.

We investigated the potential for hybridization between two endemic Hawaiian species of *Schiedea* (Caryophyllaceae), considering how variation in reproductive systems and pollination biology could influence genetic structure of the putative hybrid zone. *Schiedea*, the fifth largest plant radiation in the Hawaiian Islands, is notable for its high diversity in breeding systems and associated variation in outcrossing rates [19], [20]. Dioecious species are obligate outcrossers, while hermaphroditic species range from highly outcrossing [21] to autogamous species [22]. Species with intermediate outcrossing rates (mixed mating systems) may be hermaphroditic (e.g., *S. menziesii*) [23], gynodioecious (e.g., *S. salicaria*) [24], or subdioecious (e.g., *S. globosa*) [25], [26]. All sexually dimorphic (including gynodioecious, subdioecious and dioecious) species of *Schiedea* are wind-pollinated and occur in dry habitats [27]. In contrast, hermaphroditic species of *Schiedea*, even those occurring in dry habitats, are not adapted for wind-pollination.

Morphological and molecular traits were analyzed in a putative hybrid zone between hermaphroditic, moderately selfing *Schiedea menziesii* and gynodioecious, wind-pollinated *Schiedea salicaria*. *Schiedea menziesii* is protandrous and believed to be self-pollinated through geitonogamous pollinations [23]. No potential biotic pollinators have been observed in natural populations of this species [23]; thus, limited pollen movement leading to selfed progeny is expected to occur through wind pollination. *Schiedea salicaria* shows adaptations to wind pollination, including an abundance of pollen and smaller pollen grains than those of biotically pollinated species [27]. This species produces hermaphroditic individuals that are partially selfing and female plants that are outcrossing (Table 1). The putative hybrid nature of plants that exhibit a wide range of morphological variation resembling *S. menziesii* and *S. salicaria* was suggested by the earliest plant collectors on West Maui [19]. Thus, we hypothesized that these individuals with intermediate morphologies represent hybrids from the two hypothesized parental species rather than variants of one of the parental species. Furthermore, we hypothesized that *S. salicaria* served as the pollen parent of the putative hybrids because it is outcrossing and wind-pollinated, characteristics that should allow for more efficient pollen transfer compared to *S. menziesii*, the more selfing species. As a result of this difference in pollination, the putative hybrids are hypothesized to resemble *S. salicaria* in morphological traits due to the greater influx of genes from *S. salicaria* and selection for traits of this species. Given these hypotheses, we expected the following: (1) Plants will contain a mixture of molecular markers and morphological traits from both parents; (2) Plants will retain the maternal genome of *S. menziesii*, which will be reflected by similarity of chloroplast markers between the hybrids and *S. menziesii*; (3) Neutral nuclear molecular markers in the putative hybrids, in contrast, will be represented by a combination of those found in both parental species; (4) Morphological traits associated with wind pollination will resemble *S. salicaria* because wind has selected for characteristics that promote higher rates of pollen dispersal exhibited by *S. salicaria*. This pattern is similar to the ‘advancing wave’ dynamic described by Campbell *et al.* [15] for *Ipomopsis*, where the agent of selection is biotic, rather than abiotic.

**Table 1:** Reproductive systems of *Schiedea menziesii* and *S. salicaria*.

Species	Geographic distribution	Breeding system	Selfing level	Inbreeding depression
*S. menziesii*	W. Maui (west side) and Lanai	Hermaphroditic (one female observed in one field population, occasional females have appeared in progeny of field-collected seeds)	s = 0.607 (SD = 0.091), n = 3 population estimates (1 population sampled over 3 years; estimates based on 296–1072 progeny)	δ = 0.82
*S. salicaria*	W. Maui (east side)	Gynodioecious, populations with 12–13% females	Hermaphrodites: s = 0.398 (SD = 0.049); Females: s = 0.127 (SD = 0.038); n = 9 population estimates for both sexes (2 populations sampled at different times over 4 years; estimates based on 218–1250 progeny)	δ = 0.608 −0.870 in two experiments

Breeding systems, selfing values (s), and inbreeding depression estimates from Sakai *et al.* [25], Rankin *et al.* [23], and Weller and Sakai [24]. Selfing values for females of *S. salicaria* are below one due to biparental inbreeding.

## Results

### Morphological Traits

**Figure 1:**
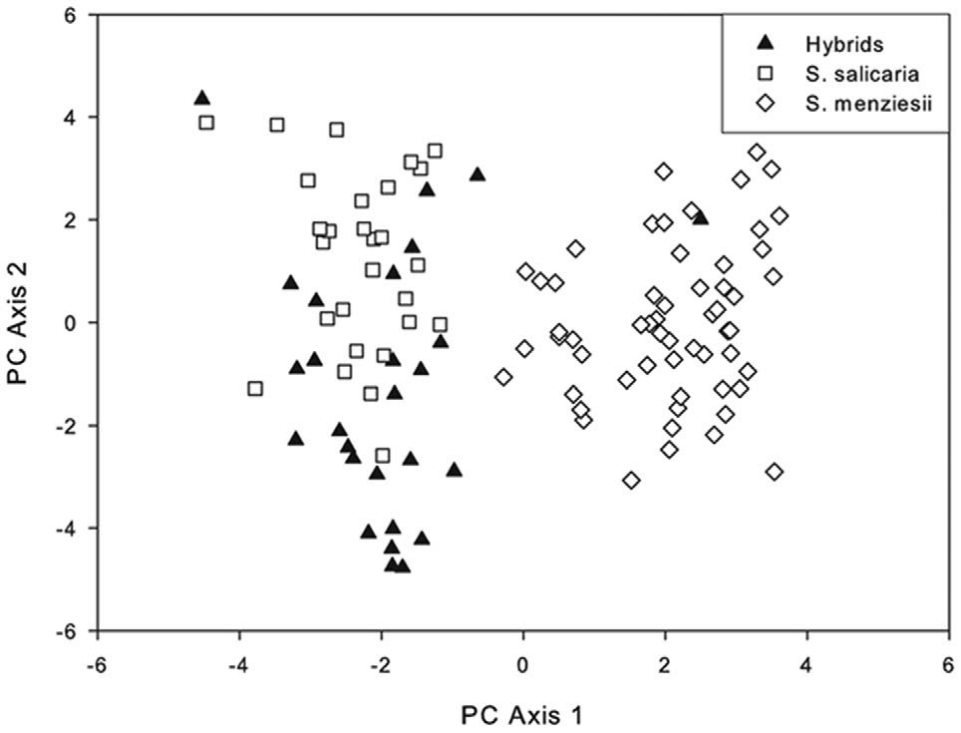
First and second principal component axes based on morphological measurements plotted for individuals of *S. menziesii* (diamonds), *S. salicaria* (squares), and hybrids (triangles).

**Figure 2:**
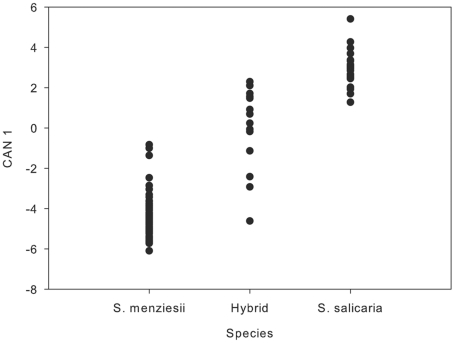
First canonical variable plotted for individuals of *Schiedea menziesii*, hybrids, and *S. salicaria*.

Hybrid individuals were variable, with some individuals possessing elongate leaves of intermediate width with a single vein, and others producing broader, weakly three-veined leaves. The first and second principal components of the multivariate analysis of morphological traits accounted for 34% and 20.5% of the observed variability (Fig. 1). The first principal component loaded most heavily on leaf and pubescence traits, as well as the relative shape of the inflorescence (narrow vs. broad due to the length of lateral branches). The second principal component loaded most heavily on internode length. Canonical discriminant analysis confirmed the major conclusions based on the principal components analysis. Traits having the highest values for the first canonical variable were leaf length and width, the number of veins in the leaves, inflorescence pubescence, pedicel length, and lateral branch length in the inflorescence. Comparison of values for the first canonical variable of the parental species and plants from the hybrid zone (Fig. 2) indicated that morphological traits in most of the hybrids were more similar in morphology to *S. salicaria* than *S. menziesii*, although four of the 14 hybrids more closely resembled *S. menziesii*.

Although differences were not significant, hybrids were intermediate between the parental species in pollen production and pollen size, with values for both traits closer to *S. salicaria*, the wind-pollinated parent. Based on seven hybrid individuals, pollen production per flower for hybrids averaged 14,645 pollen grains per flower (SE = 1,113), compared to values of 14,756 (SE = 1,689) for *S. salicaria*, and 13,935 (SE = 1,645) for *S. menziesii* [27]. Pollen size of the hybrids averaged 34.0 µm (SE = 0.47), compared to 33.4 µm (SE = 1.76) for *S. salicaria* and 34.8 µm (SE = 0.71) for *S. menziesii*. Average pollen fertility of the hybrids was 95.9% (SE = 1.29).

The hybrid zone included plants that were female. Of the 25 flowering plants surveyed for sex expression in the field, 7 (28%) were female, not significantly different from the 12–13% frequency of females observed in populations of *S. salicaria* [28]. *Schiedea menziesii* is primarily hermaphroditic in the field. Only one out of several hundred plants examined in three field populations was female.

### Nuclear Microsatellites

Despite the rarity of these taxa, a substantial amount of genetic variation was detected within taxa and populations (Table 2). Significant deviations from Hardy-Weinberg equilibrium were detected for locus SA30 in *S. salicaria* population 842, *S. menziesii* population 950, and the hybrids, and for locus SA31 in *S. menziesii* population 950 and the hybrids. Possible null alleles were detected in several populations for locus SA30 (*S. menziesii* 849, 949, 950; *S. salicaria* 842; hybrids), but this locus did not differ significantly in allele frequencies among the parents or hybrids (Table 3). Additional null alleles were detected at locus SA04 in population *S. menziesii* 950 and at locus SA06 in population *S. salicaria* 842. Over 85% of the loci were polymorphic in both species and their hybrids, and expected heterozygosity was high in all taxa (*S. salicaria*: *H_o_* = 0.45, *H_e_* = 0.53; *S. menziesii*: *H_o_* = 0.35, *H_e_* = 0.45; hybrids: *H_o_* = 0.39, *H_e_* = 0.49; Table 2). The hybrid population contained more alleles on average (*A* = 3.67; *A_p_* = 4.00) than *S. salicaria* (*A* = 3.56; *A_p_* = 3.88) or *S. menziesii* (*A* = 3.26; *A_p_* = 3.64). *Schiedea menziesii* and *S. salicaria* shared 28 alleles, and the hybrids harbored 22 of these. The hybrids shared four alleles with *S. menziesii* that were not present in *S. salicaria*, and two alleles shared between the hybrids and *S. salicaria* were not found in *S. menziesii*. Five rare alleles were unique to the hybrids (mean frequency = 0.064). Despite the lack of high frequency species-specific alleles, *S. menziesii* and *S. salicaria* exhibited significantly different allele frequencies at six of the nine loci (*F_ST_* = 0.117; P<0.0001; Table 3).

**Table 2:** Genetic diversity at nine nuclear microsatellite loci in populations of *Schiedea menziesii*, *S. salicaria* and hybrids.

Taxon	Population	*N*	*A*	*A_p_*	*P*	*H_e_*	*H_o_*
*S. menziesii*	849	11.89	3.33	3.62	88.89	0.468	0.402
	949	7.56	2.89	3.43	77.78	0.414	0.305
	950	15.89	3.56	3.88	88.89	0.465	0.342
	Mean	11.78	3.26	3.64	85.19	0.449	0.350
*S. salicaria*	842	18.78	3.89	3.89	100.00	0.573	0.473
	853	5.89	3.22	3.86	77.78	0.487	0.422
	Mean	12.33	3.56	3.88	88.89	0.530	0.448
Hybrid		13.89	3.67	4.00	88.89	0.492	0.391

Shown are sample size averaged over loci (*N*), mean number of alleles per locus (*A*) and per polymorphic loci (*A_p_*), percentage of polymorphic loci (*P*; 95% level), the expected proportion of heterozygous loci per individual (*H_e_*) and observed heterozygosity (*H_o_*).

**Table 3:** Locus-by-locus analysis of differences in allele frequencies between *Schiedea menziesii*, *S. salicaria* and hybrids.

Locus	MENZ-SAL		HYB-MENZ		HYB-SAL	
	*F_ST_*	P	*F_ST_*	P	*F_ST_*	P
SA04	**0.151**	**0.0002**	**0.112**	**0.01**	0.003	0.35
SA36	**0.221**	**<0.0001**	NA	NA	**0.139**	**0.006**
SA05	0.018	0.07	0.034	0.06	0.026	0.11
SA30	0.008	0.41	0.008	0.61	0.003	0.52
SA15	**0.073**	**0.008**	0.033	0.12	0.002	0.413
SA38	**0.276**	**<0.0001**	**0.146**	**0.003**	0.002	0.402
SA32	**0.107**	**0.002**	0.045	0.10	0.038	0.06
SA06	**0.286**	**<0.0001**	0.009	0.52	**0.166**	**0.003**
SA31	0.022	0.221	0.009	0.44	0.047	0.15
All	0.117	<0.0001	0.049	0.004	0.044	0.005

MENZ, *S. menziesii*; SAL, *S. salicaria*; HYB, hybrids. *F_ST_* and P-value for each comparison are reported. P-values were determined from 10,000 permutations of the data. Significant values are indicated in bold.

**Figure 3:**
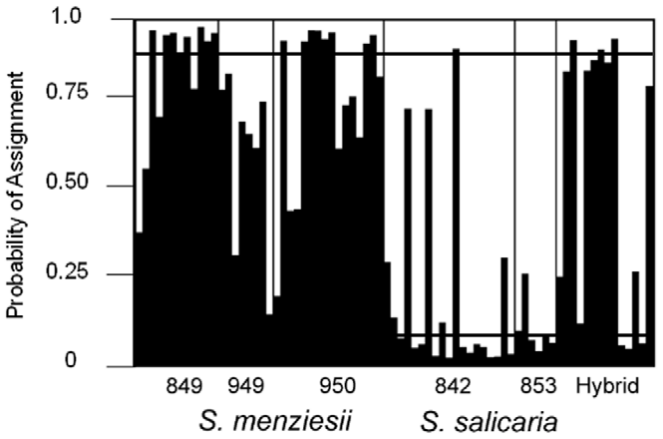
Assignment of samples of *S. menziesii*, *S. salicaria*, and hybrids to two clusters (K = 2) using a Bayesian clustering algorithm in STRUCTURE with the nuclear microsatellite data. For each individual the probability of assignment to the two clusters is indicated. A cut-off of 90% probability of assignment to a single cluster was used to assign individuals; this cut-off is indicated by the horizontal lines at the top (for black bars) and bottom (for white bars) of the plot. Black bars correspond to the primarily–*S. menziesii* cluster, and white bars to the primarily-*S. salicaria* cluster. Individuals with mixed ancestry exhibit a combination of black and white bars with neither bar reaching 90% probability.

No evidence of linkage disequilibrium was detected in the hybrid population, but we did find significant *F_ST_* values (P<0.05) for four of the six loci that also exhibit significantly different allele frequencies in the parental species (Table 3). The Bayesian assignment of population ancestry based on only two groupings followed species designations for many *S. menziesii* and *S. salicaria* individuals (Fig. 3). Sixteen of the 36 samples of *S. menziesii* were assigned to a single cluster and all had probabilities of assignment greater than 91% (see black bars in Fig. 3). None of the eight samples from population 949 could be assigned to a single cluster, but this may reflect intraspecific variation that is also apparent in the cpDNA data set (see results below). Sixteen of the 25 samples of *S. salicaria* were also assigned to a single cluster with greater than 91% probability, and this cluster was distinct from *S. menziesii* (see white bars in Fig. 3). One *S. salicaria* plant out of 20 samples in population 842 was assigned to the *S. menziesii* cluster with a probability of 92%. Six hybrid samples were assigned to a single cluster (three to each parental cluster) with a probability of at least 91%, whereas the remaining eight hybrid plants exhibited shared ancestry between the two clusters (Fig. 3).

### Chloroplast Markers

**Figure 4:**
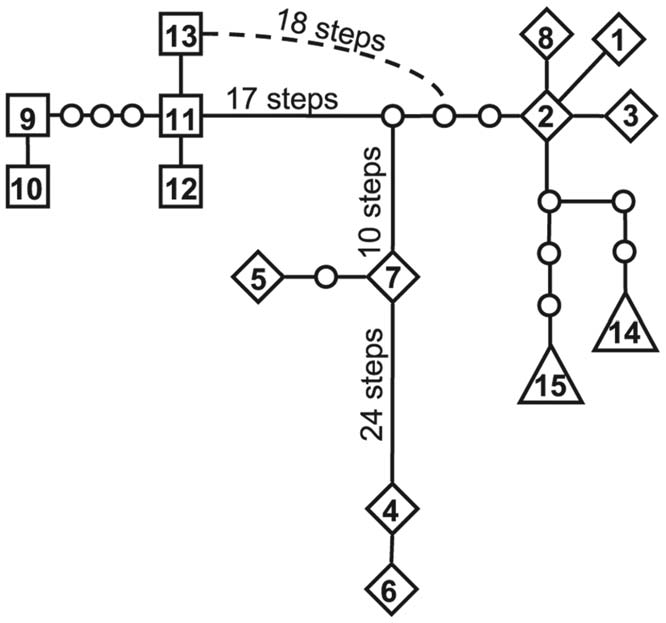
Haplotype network constructed from multilocus chloroplast sequences observed in *Schiedea menziesii*, *S. salicaria* and hybrids. Gaps were included in the analysis. Observed haplotypes are numbered and indicated as diamonds (*S. menziesii*), squares (*S. salicaria*), or triangles (hybrids). Small circles represent unobserved inferred haplotypes. The line connecting each haplotype indicates one mutational change, except where otherwise indicated. Closed connections among haplotypes indicate an alternative relationship between haplotypes. The 24 steps between haplotypes 7 and 4 include a 23 base indel in *ndh*J-*trn*F, the 10 steps between an inferred haplotype and haploytpe 7 include a 10 base indel in *psb*M-*trn*D, and a 5 base indel in *rpL*16 distinguishes *S. menziesii* and hybrid haplotypes from those of *S. salicaria*.

The five loci exhibited different levels of variation within each taxon (Table S1). In the concatenated data set, 15 unique multilocus chloroplast haplotypes were identified, including eight in *S. menziesii*, five in *S. salicaria*, and two in the hybrids (Table 4). No hybrid individual contained a haplotype that was identical to either parental species, but the hybrid haplotypes were more similar to *S. menziesii* than to *S. salicaria*, including having single locus haplotypes identical to those of *S. menziesii* at four of the loci (*rp*S16, *pet*N-*psb*M, *psb*E-*pet*L, *trn*L microsatellite) and differing by a minimum of four steps from *S. menziesii* haplotype 2 (Fig. 4). This topology is robust to different methods of gap coding and analysis as similar results were produced when we employed multistate gap coding [29] and maximum parsimony analyses [30] (results not shown).

**Table 4:** Chloroplast diversity in populations of *Schiedea menziesii*, *S. salicaria*, and hybrids.

Taxon	Population	*N_no gaps_*	*N_gaps_*	*H* (SD)	π (SD)
*S. menziesii*	849	2	1 (0.21)	0.527 (0.064)	0.0001
			2 (0.36)		(0.00002)
			3 (0.43)		
	949	4	4 (0.64)	0.643 (0.184)	0.0030
			5 (0.12)		(0.00012)
			6 (0.12)		
			7 (0.12)		
	950	2	1 (0.31)	0.325 (0.125)	0.0006
			2 (0.44)		(0.00024)
			4 (0.19)		
			8 (0.06)		
	Total	6		0.640 (0.067)	0.0009
					(0.00013)
*S. salicaria*	842	1	9 (0.95)	–	–
			10 (0.05)		
	853	1	11 (0.5)	–	–
			12 (0.17)		
			13 (0.33)		
	Total	1		–	–
Hybrid		1	14 (0.92)	–	–
			15 (0.08)		

The number of distinct haplotypes without considering gaps (*N_no gaps_*), distinct haplotypes when considering gaps and their relative frequency in the population (*N_gaps_*), haplotype diversity and standard deviation (*H*), and nucleotide diversity and standard deviation (π) are reported. Haplotype numbers for *N_gaps_* correspond to those depicted in the network in Fig. 4.

Gene diversity was highest in *S. menziesii* (*H* = 0.640), with *S. salicaria* and the hybrids exhibiting no nucleotide diversity (Table 4). However, variation was observed in *S. salicaria* and the hybrids in insertions/deletions. The two *S. salicaria* populations did not share haplotypes, although the haplotypes in these two populations were distinct from hybrid and *S. menziesii* haplotypes. Within *S. menziesii*, populations 849 and 949 also did not share haplotypes, but population 950 shared haplotypes with the other two *S. menziesii* populations (Table 4). AMOVA indicated that *S. menziesii* and *S. salicaria* were genetically distinct (*F_ST_* = 0.68; P<0.0001) and that the hybrids were also distinct from both parents (*S. menziesii*: *F_ST_* = 0.29, P = 0.002; *S. salicaria*: *F_ST_* = 0.97, P<0.0001).

## Discussion

The dynamic landscape of oceanic islands, where active volcanoes continue to generate new land masses, suggests a wealth of opportunity for interspecific hybridization through the erosion of ecological barriers or the persistence of hybrid individuals in habitats separated from parental species. Indeed, instances of historical and on-going hybridization are increasingly reported [31], [32]. On the Hawaiian Islands, 210 instances of interspecific hybridization spanning 59 genera have been documented [32]. Among these are the Hawaiian silversword alliance [33]–[37], *Cyrtandra* [38], *Metrosideros* [39], *Portulaca* [40], *Rubus* [41], and *Scaevola* [42]. In addition to the *S. menziesii- S. salicaria* hybrid zone discussed here, Willyard *et al.* [43] uncovered several instances of cryptic chloroplast introgression throughout *Schiedea*. As expected of recently evolved systems, many of these hybridization events occur between species that are not widely divergent genetically [44], and ecological barriers are typically stronger than genetic barriers to gene flow [39], [45]. Given the increased detection of interspecific hybridization in continental and island plant systems, a more detailed understanding of the adaptive nature of hybridization [46] is warranted. Studies that utilize multiple sources of data, as we have here, serve as the best tests of the dynamics of hybridization and the adaptive potential inherent in hybrids.

### Status of the Putative Hybrids

Plants exhibiting a wide range of morphological variation resembling *S. menziesii* and *S. salicaria* were found on West Maui by the earliest plant collectors and have been suggested to be putative hybrids between these species [19] or variants of *S. salicaria* [47]. The combination of morphological traits and nuclear and chloroplast genetic markers described in the present study suggests that all plants from Halepohaku, the putative hybrid zone, are hybrids of *S. menziesii* and *S. salicaria*, rather than variants of *S. salicaria sensu stricto* [47] or a variety of *S. salicaria* [48]. Whereas the two parental species are morphologically and genetically distinct, the hybrid population exhibits evidence of ancestry from both parents. The hybrids most closely resembled *S. salicaria* in inflorescence and vegetative traits (Figs. 2, 3), but they have chloroplast genomes that are more similar to those in *S. menziesii* (Fig. 4). Although few species-specific alleles were found at the nuclear microsatellite loci, there is also evidence in these data to suggest a hybrid origin for these plants. The parental species are significantly differentiated at six of the nine loci examined (*F_ST_* = 0.117; P<0.05), and the hybrids exhibit significant differences from just one parent at four of these loci (Table 3), suggesting that the other parent was the predominant donor of alleles at those loci. If the purported hybrids were variants of a single species, then they would be expected to show the same patterns of allelic differentiation when compared to conspecific populations. The hybrids also have a greater number of alleles per locus than either parental species, consistent with the expectation that both species have contributed nuclear alleles to the hybrid zone. Bayesian assignment of hybrid plants to the two clusters, which roughly correspond to the two parental species, also points to a hybrid origin for many of the plants in this population. Welch and Rieseberg [49] found a similar lack of species-specific alleles, but divergent allele frequencies, in the diploid parents of the hybrid species, *Helianthus paradoxus*. According to Chapman and Burke [44], low levels of genetic divergence between parental species are more likely to result in homoploid hybrids than allopolyploids after interspecific hybridization. Although chromosome counts have not been done for these species of *Schiedea*, the high level of fertility between hybrid zone plants and *S. salicaria* and between *S. menziesii* and *S. salicaria*, suggests that there are not ploidal differences between the parents and plants from the hybrid zone (S. Weller and A. Sakai, unpubl. data). No more than two alleles were ever found at the microsatellite loci, which also suggests the hybrids are diploid. Thus, the lack of species-specific alleles at nuclear loci in *S. menziesii* and *S. salicaria* is consistent with findings in other hybrid systems involving closely related parental taxa.

The hybrid population appears to be a naturally occurring contact zone between *S. menziesii* and *S. salicaria*, given that human encroachment in the area is limited. In light of Hillebrand's early collections, the *S. menziesii - S. salicaria* hybrid zone is likely a stable population that has existed for more than a century. Although the nuclear loci were not sufficiently variable to differentiate among early and late generation hybrids, the wide range of morphological and genotypic variability among the hybrids suggests these individuals are not first generation hybrids. Viable hybrids of *S. menziesii* and *S. salicaria* form readily in the greenhouse and are morphologically similar to naturally occurring hybrids (S. Weller & A. Sakai, unpublished data). The high fertility of the hybrids under these conditions indicates that there are unlikely to be intrinsic barriers to gene exchange among the hybrids in the field, consistent with the hypothesis that plants in the hybrid zone are F_2_ or later generation hybrids. Additionally, we did not find significant linkage disequilibrium among any of the loci in the hybrid population, which further suggests that this is an older stable hybrid population in which certain allelic combinations have been broken up and a range of genotypes are now present in the population. Thus, this hybrid zone is most similar to the stable zones predicted when hybrids have equal or greater fitness than their parents [5], [6].

### Asymmetric Gene Flow Occurs from *S. salicaria* to *S. menziesii*

Hybrid zones can be generated from unidirectional gene flow from one species to another, or through bidirectional gene flow with both species serving as maternal and paternal parents. The direction of gene movement in hybrid zones has important implications for understanding the evolutionary potential and sustainability of hybrid progeny. For example, both parents have contributed to the cytoplasmic diversity of the hybrid sunflower *Helianthus deserticola*, leading to high levels of differentiation among populations [50]. In the polyploid *Tragopogon* complex, *T. miscellus* exhibits different floral morphology depending on whether *T. dubius* or *T. pratensis* is the maternal parent [51]. In contrast, other studies of natural hybrid zones have demonstrated unidirectional gene flow [14], [52], [53]. Actually, instances of asymmetric gene flow in hybrid zones are commonly documented in other natural systems [3], including Hawaiian taxa. For example, Friar *et al.* [35] identified ongoing hybridization from Hawaiian *Dubautia ciliolata* to *D. arborea* leading to the formation of an active hybrid zone. However, there was little evidence of introgression into parental populations [35]. Randell *et al.* [41] also found asymmetrical hybridization between *Rubus hawaiiensis* and *R. rosifolius* on Maui based on the presence of shared cpDNA haplotypes between the hybrids and *R. rosifolius* but not *R. hawaiiensis*. Howarth and Baum's [42] study of *Scaevola* demonstrates that homoploid hybrid speciation may be particularly common in island systems where congeneric species are often not strongly diverged. We have also found evidence that hybridization occurs readily between *S. menziesii* and *S. salicaria* and that gene flow in this natural hybrid zone has been asymmetrical. Assuming that the cpDNA genome is maternally inherited in *Schiedea*, an assumption that is supported across many flowering plant families [54], [55] as well by our sequence data on maternal families (results not shown), the cpDNA data are most consistent with *S. menziesii* being the seed parent of the extant hybrids. Although haplotypes were not shared between species or with the hybrids, the hybrid haplotypes were more genetically similar to those of *S. menziesii* from which they differed a minimum of four mutational steps from *S. menziesii* haplotypes on the central ridge of Lihau (849; Fig. 4 haplotypes 1–3) and those from the gulch below this ridge (950; Fig. 4 haplotypes1–3 and 8), but they differed at least 24 steps from *S. salicaria* haplotypes (Fig. 4 haplotypes 9–13). This implies that the pollen parent has been *S. salicaria*, and this is consistent with the occurrence of wind pollination in *S. salicaria* and its absence in *S. menziesii* [27].

**Figure 5:**
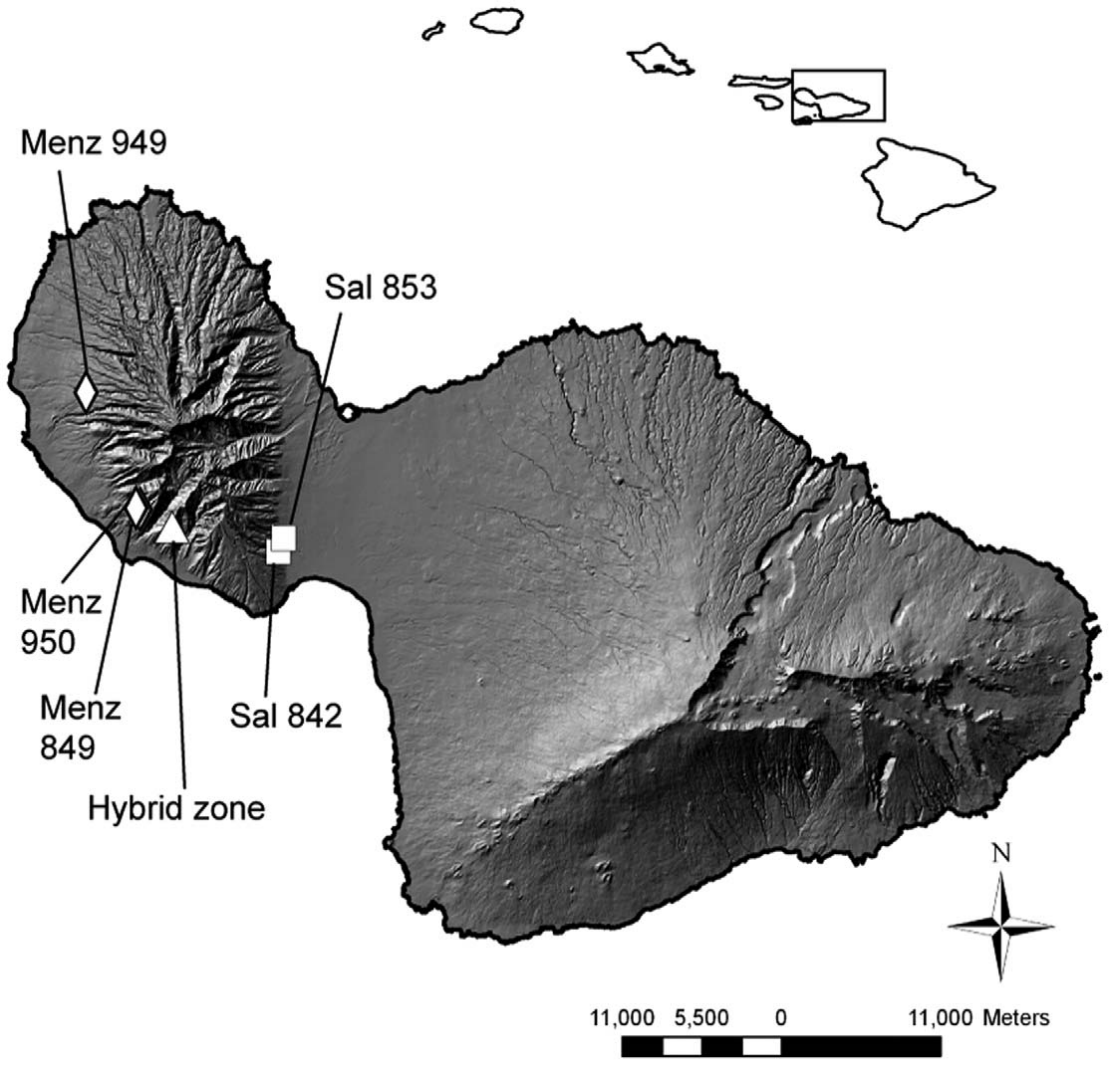
Locations of sampled populations on West Maui. *Schiedea menziesii*, diamonds; *S. salicaria*, squares; hybrids, triangles. The inset map shows the location of Maui relative to the other Hawaiian Islands.

The conditions that facilitated the initial formation of hybrids certainly required that *S. menziesii* and *S. salicaria* be in closer physical contact than they are today, and this likely occurred in conjunction with past environmental and/or climatic changes on the Hawaiian Islands [56], [57]. For example, the island of Maui was part of Maui Nui, an island that reached a maximum size of 14,000 km^2^ [57] during the Pleistocene. It is expected that contact between the parental species occurred during this time as they expanded their ranges to new areas of Maui Nui. Presently, the parental species are not in close physical contact. *Schiedea menziesii* occurs in shrublands situated on ledges and cliffs from sea level up to 670 m, while *S. salicaria* is found in shrublands along steep slopes from 180 to 670 m. The extant hybrid zone is in an area closer to the range of *S. menziesii*, and other hybrid zones have also been noted in this area on West Maui [19]. Trade winds blow across the Hawaiian Islands from a northeasterly direction, but wind direction on the scale necessary for pollen movement is variable and depends upon local topography (S. Weller & A. Sakai, pers. observ.). While *S. salicaria* populations are currently situated to the east of *S. menziesii*, they are too distant to serve as the source of pollen. Thus, we suggest that populations of *S. salicaria* likely occurred closer to the hybrid zone in the past, and this permitted interspecific hybridization to occur. Contact between the parental species may have been limited geographically, given that that *S. menziesii* from the Hahakea population 949, located 10.3 km north of the hybrid zone (Fig. 5) is genetically distinct with cpDNA haplotypes that are nearly as distinct from haplotypes in conspecific populations 849 and 950 as they are from *S. salicaria* and the hybrid haplotypes. Population 949 also exhibited genetic differences in the nuclear data and could not readily be assigned to the *S. menziesii* cluster in the Bayesian analysis.

We found no evidence of introgression of *S. menziesii* plastid haplotypes into extant *S. salicaria* populations. However, there may be some evidence of bidirectional gene flow within the nuclear data set. Based on the Bayesian assignment analysis, one of the *S. salicaria* samples has a higher probability of ancestry from *S. menziesii*, and 29 other parental samples (20 of *S. menziesii* and 9 of *S. salicaria*; Fig. 3) exhibit shared ancestry. While this could be due to low polymorphism and a lack of species-specific alleles at these loci, many individuals were confidently assigned to a single cluster, and the species have significantly different allele frequencies (Table 3). Additional nuclear data from more variable loci are needed to validate this inference, but these data suggest cryptic remnants of gene flow from *S. menziesii* into *S. salicaria*. Similar patterns of heterogeneous gene flow across the genome have been reported in *Dubautia* [37]. The widespread lack of chloroplast introgression from *S. menziesii* into *S. salicaria* could be due to selection against individuals with this cytonuclear combination or pollen-swamping by *S. salicaria* due to its greater pollen production and more effective means of pollen dispersal compared to *S. menziesii*.

Currat *et al.* [3] predict that unidirectional cytoplasmic introgression should occur from the local species to the invading species. Early on, bidirectional gene flow could have occurred during the initial contact between parental species even in the face of greater pollen production by *S. salicaria* if *S. menziesii* outnumbered invading *S. salicaria* plants. If F_1_ hybrids containing cpDNA genomes of *S. salicaria* subsequently served as pollen parents in backcrosses to *S. menziesii* and continued to increase in frequency, then all traces of *S. salicaria* serving as the seed parent would be lost. It is likely that the extant hybrids do not resemble *S. salicaria* in their chloroplast genome because they are late-generation hybrids and there has been widespread pollen-swamping by *S. salicaria* or hybrids that resemble this species. By having a distinct advantage in greater pollen production than *S. menziesii*, hybrids that were more like *S. salicaria* would have been more likely to swamp *S. menziesii*, thereby yielding hybrids with cpDNA haplotypes that are more similar to *S. menziesii*.

The absence of identical haplotypes in the hybrids and *S. menziesii* can be explained by 1) unsampled *S. menziesii* populations, including extinct populations, containing those haplotypes, 2) origin from *S. menziesii* haplotypes followed by mutational changes in the chloroplast loci since the formation of the hybrid zone, or 3) the presence of heteroplasmy and recombination within the chloroplast genome, which have been demonstrated for mitochondrial genomes in other members of the Caryophyllaceae [58]. We do not believe that the hybrid haplotypes are recombinants because there is little homoplasy in the haplotype network and none associated with the hybrid haplotypes. Additionally, if they were recombinants, we would expect the hybrids to exhibit variation in how similar they are to each of the parents at different loci. Instead, we see a greater similarity to *S. menziesii* at six of the seven loci, suggesting a direct origin from this species rather than recombination. Evaluation of the other two hypotheses requires comparison of additional genetic markers to quantify genome-wide variation between the hybrids and the parental species.

### Wind Pollination Favors Traits of *S. salicaria* in the Hybrid Zone

When hybridization occurs between selfing and outcrossing species, traits of the outcrossing species are expected to be favored among hybrids. Moreover, when there are differences in pollination mechanism, it is expected that selection will favor the species with more effective pollen dispersal. Because *S. salicaria* disperses pollen via wind while *S. menziesii* shows no indication of wind pollination [27], we hypothesized that selection should favor morphological traits most similar to *S. salicaria* in the hybrids as a result of pollen-swamping by this species. If wind is the selective agent in the hybrid zone, traits that promote more effective pollen transfer will be favored. Thus, in each generation, *S. salicaria* traits promoting wind pollination (i.e., small pollen size, greater pollen production, inflorescence traits) should increase in frequency in the hybrid zone because individuals with these traits are most likely to serve as pollen donors. Other *S. salicaria* traits controlled by nuclear genes should also increase as wind pollination favors the nuclear genome of *S. salicaria*. We found a high degree of similarity between the hybrids and *S. salicaria* in the traits associated with wind pollination, including greater pollen production, smaller pollen size, more flowers per inflorescence, and the details of inflorescence shape (Figs. 1, 2). Thus, these data are consistent with evolution of morphological traits associated with wind-pollination among the hybrid plants.

Male-sterility is controlled by nuclear genes in *Schiedea* [59], and as a result, breeding system traits were also expected to be influenced by hybridization between *S. menziesii* and *S. salicaria*. The presence of unisexual flowers is often seen in conjunction with wind pollination [60]–[63]. In *Schiedea*, once male-sterility alleles have been introduced into a population, wind pollination may be necessary for females to become established in populations because of pollinator limitation [27], [61]. We predicted that some hybrid individuals would exhibit male sterility as a result of gene flow from gynodioecious *S. salicaria* to hermaphroditic *S. menziesii*. The occurrence of male sterility in the hybrid zone (28% females) suggests that gene flow from *S. salicaria* has prominently shaped the reproductive system of the hybrid zone. In conjunction with the morphological adaptations to wind pollination, females may have a selective advantage in the ecological setting of the hybrid zone. In contrast, females are very rare in the field for *S. menziesii*, presumably because of the absence of both pollinators and traits favoring wind pollination [23], [27]. Hybrids between *S. menziesii* and *S. salicaria* may be evolving towards more effective mechanisms for wind pollination and outcrossing, including the occurrence of females, in light of the high levels of inbreeding depression (Table 1) found in populations of both parental species [23], [25], and the occurrence of high levels of heritable variation for traits associated with wind pollination [64], [65].

### Conclusions

We have documented the presence of an interspecific hybrid zone between two species of *Schiedea* on West Maui and found evidence of unidirectional cytoplasmic gene flow contributing to the diversity of the current hybrid population. Although bidirectional gene flow may have occurred in the past, as evidenced by mixed ancestry of some individuals from the parental species, we suggest that the selective advantage of wind pollination in the hybrid zone has driven phenotypes towards *S. salicaria*, the wind pollinated species. Because *S. menziesii* is not adapted to wind pollination and seed dispersal occurs over very short distances in all *Schiedea* species, introgression from *S. menziesii* is unlikely to alter the breeding system of *S. salicaria* [64]. However, the hybrid zone could influence the evolution of dimorphism in *S. menziesii* by introducing genes from *S. salicaria* associated with male sterility and wind pollination if these plants come in contact with hybrids of *S. salicaria* in the future. Hybridization has facilitated the evolution of dimorphic breeding systems from hermaphroditism in other species [66]. Recent phylogenetic analyses of *Schiedea* have revealed widespread hybridization events leading to chloroplast capture [43]. Given the dynamics of the hybrid zone between *S. menziesii* and *S. salicaria* uncovered here, interspecific hybridization may have the potential to promote breeding system evolution in other species of *Schiedea*, just as it has promoted radiations of other notable Hawaiian endemic groups [34], [42].

## Materials and Methods

### Study Organisms

*Schiedea menziesii* and *S. salicaria* are rare shrubs endemic to the Hawaiian Islands and inhabiting remnant dry shrubland of the West Maui Mountains [19], [67]. *Schiedea salicaria* is currently known only on the eastern side of West Maui. One historic collection of *S. salicaria* near Lahaina on the west side of West Maui is likely to represent a hybrid with *S. menziesii* [19]. Both *S. menziesii* and *S. salicaria* occur on dry, north-facing steep slopes at 30–670 m and 180–670 m elevation, respectively [19]. Both species were more widespread historically and one population of *S. menziesii* on Lana'i has been extirpated. Leaves of *S. menziesii* are typically falcate and linear to linear-lanceolate while those of *S. salicaria* are shorter, symmetrical, and elliptic [19]. *S. salicaria* leaves have a single vein, while leaves of *S. menziesii* almost always have three veins running the length of the leaf. Inflorescences of both species are similar, although those of *S. menziesii* are slightly more laterally compressed and have fewer, larger flowers. Flowers are apetalous and appear during the wetter winter months when most plant growth occurs. Based on wind tunnel studies, *S. salicaria* is wind-pollinated, while *S. menziesii* is not adapted to wind pollination and has larger pollen grains and reduced pollen production compared to *S. salicaria* [27]. Hermaphrodites of both species have substantial levels of self pollination (s = 0.398–0.607, Table 1) [23], [24]. *Schiedea salicaria* is gynodioecious with 12–13% females [25], [28] while *S. menziesii* has previously been considered hermaphroditic [67]. Females of *S. menziesii* have been produced from field-collected seed, however, and a single female individual has been observed in a natural population (Table 1; T. Culley, pers. obs.). Sex in *Schiedea* is under nuclear control with male sterility determined at a single locus [59]. Females are homozygous recessive (*hh*) and hermaphrodites are either heterozygous (*Hh*) or homozygous dominant (*HH*). Vigorous hybrids have been produced between the two species by hand-pollinations under greenhouse conditions (S. Weller & A. Sakai, unpublished data). Putative natural hybrid populations have also been documented on West Maui [19]. Plants within these putative hybrid populations exhibit a wide range of morphological variation in vegetative traits.

The hybrid zone sampled for this study occurs on Halepohaku, on the southwest side of West Maui between the ranges of the two parental species (Fig. 5). Sex ratios were recorded in February, 1995. All plants located in this area appeared to be of hybrid origin based on their morphological traits. *Schiedea salicaria* was sampled from two populations on the east side of West Maui (Fig. 5; Table 5), the only area where *S. salicaria* is known to occur. Three populations of *S. menziesii* were sampled, all located north of Halepohaku (Fig. 5; Table 5).

**Table 5:** Populations of *Schiedea menziesii*, *S. salicaria*, and hybrids sampled in the current study.

Taxon	Population	Location (all W. Maui)	Morphology	Nuclear Microsatellites	Chloroplast Markers
*S. menziesii*	849	Lihau Ridge	18	12	14
	949	Hahakea Gulch	13	8	8
	950	Gulch N of Lihau Ridge	25	16	16
*S. salicaria*	842	Pu'uhona	28	20	19
	853	Ka'onohua Gulch	NA	6	6
Hybrid		Halepohaku, ridge between Ula 'ula and Ko'ai	14	14	13

The population name (accession number), location on West Maui, and number of individuals included in the morphological, microsatellite, and chloroplast data sets are reported.

### Ethics Statement

Individuals of both species and hybrids sampled for molecular analysis originated as field-collected seeds or cuttings from several different populations (Fig. 5; Table 5; vouchers at US) and were grown in the greenhouse at the University of California, Irvine. All necessary permits were obtained for the described field studies. These permits covered the collection of *S. menziesii* plants in the Lihau Natural Area Reserve on W. Maui (granted by B. Gagne), putative hybrid plants from the Pu'u 'Ula'ula area on W. Maui collected by S. Perlman and K. Wood (granted by the State of Hawaii), and *S. salicaria* plants (granted by Wailuku Agribusiness) prior to its listing as endangered. Permission to study a population of *Schiedea menziesii* was granted by J. Falconer at the Pioneer Mill Company on Maui, on land owned by Pioneer Mill.

### Morphological Analyses

Traits measured in the greenhouse were those distinguishing the two species [19], including inflorescence length, number of inflorescence nodes, length of the lateral inflorescence branches, total number of flowers in the inflorescence, internode lengths of vegetative portions of flowering branches, leaf length, leaf width, and leaf curvature, number of leaf veins and degree of leaf pubescence, sepal length and extent of sepal pubescence, and pedicel length and pedicel pubescence. Principal components analysis (PCA) [68] was used to detect patterns of similarity between hybrid individuals and the presumed parental species. Additionally, canonical discriminant analysis (Proc Candisc in SAS) was used to calculate raw canonical coefficients of the first canonical variable for the parental species. Hybrids were not included in this latter calculation because their inclusion could obscure differences between the parental species. Subsequently, these coefficients were used to calculate the first canonical variable for the parental species and the hybrids.

Pollen production of each putative hybrid individual was measured by placing undehisced anthers of 2–6 flowers per genotype in a lactophenol-aniline blue solution [27]. Five to seven genotypes per species were used to estimate pollen production and pollen size. Ten hemacytometer counts per genotype were used to estimate pollen production per flower, and genotype averages were then averaged to obtain values for each of the species and plants from the hybrid zone. Pollen size was estimated by measuring the diameter of 50 pollen grains per sample. Individual means of each plant were used as replicates in the hybrid population, and compared to values for the parental species reported earlier [27]. Fully formed pollen grains that stained dark blue in aniline blue were assumed to be viable.

### Genetic Analyses

Leaf tissue was collected from plants maintained in the greenhouse. DNA was extracted from frozen or silica-dried tissue using either a modified CTAB-based technique of Doyle and Doyle [69] at the University of Cincinnati or with the DNeasy Plant Mini Extraction Kit (Qiagen, Valencia, CA, USA) at the University of South Dakota. DNA was stored at −20°C until further analysis. We genotyped individuals at nine nuclear microsatellite loci and two chloroplast microsatellite loci, and sequenced individuals for five chloroplast regions.

### Nuclear Microsatellites

Nine of the 12 microsatellite primers originally developed for *S. adamantis* [70] were used in the current study of *S. salicaria* and *S. menziesii*. These primers amplified di- (*SA30*), tri- (*SA04*, *SA05*, *SA15*, *SA32*, *SA36*, *SA38*), and pentanucleotide (*SA06, SA31*) repeats consistently across all populations. DNA samples were analyzed in multiplexed reactions which also included fluorescent labeling of the 5′ end of each fragment during the PCR process [70]. Each sample was evaluated using two different primer groups: Group 1 (*SA04, 05, 15, 30, 36, 38*) and Group 2 (*SA06, 31, 32*). PCR was performed in 10 µL reaction volumes with the Qiagen Multiplex Kit (Qiagen Inc., Valencia, CA) as follows: 5 µL Multiplex PCR Master Mix, 1 µL 10× Primer Mix (each primer at 2 µM), 3.8 µL dH20, and 0.2 µL DNA. The thermal cycler program consisted of 95°C for 15 min, followed by 30 cycles of 94°C for 30 s, 57°C for 45 s, and 72°C for 45 s, and then with 8 cycles each of 94°C for 30 s, 53°C for 45 s, and 72°C for 45 s. A final extension consisted of 72°C for 10 min. PCR was conducted at the University of Cincinnati and PCR products were run on a 3730 XL Sequencer at the Cornell University BioResource Center using the LIZ 500 (Applied Biosystems, Foster City, CA, USA) internal size standard with resulting data analyzed in Genemarker (SoftGenetics LLC, State College, PA, USA).

We tested for the presence of null alleles using Micro-Checker [71]. Genetic variability at the microsatellite loci was characterized within the two parental species and putative hybrids by the mean number of alleles per loci (*A*), percentage of polymorphic loci (*P*; 95% level), observed heterozygosity (*H_o_*), and the expected proportion of heterozygous loci per individual (*H_e_*) using the GDA software package [72]. No alleles were fixed in either of the parental species, thus we looked for evidence of admixture in allele frequencies of the putative hybrid population. We tested for significant differences in allele frequencies at each locus between the hybrids and each of the parental species using AMOVA [73] to assign parental origin of the predominant alleles at each microsatellite locus. These analyses were conducted on allele frequencies and assuming an infinite alleles model (i.e., *F_ST_*) in Arlequin v. 3.11 [74]. Significance of *F_ST_* was tested through 10,000 permutations of the data. A hybrid population may be expected to exhibit allele frequencies that are more similar to one parent at loci that are distinct between the parental species. Furthermore, when looking across all loci, a mosaic of differentiation is expected in the hybrid zone. By contrast, if the putative hybrids are variants of one parental species, then we expect allele frequencies of the hybrids to only be distinct from the non-parental species. We also tested for linkage disequilibrium (LD) within the putative hybrid population using the Burrows composite estimate based on genotypic frequencies calculated in Popgene v. 1.31 [75]. Recently hybridized populations should exhibit strong LD if they are derived from two gene pools with differing allele frequencies. Linkage disequilibrium should decay with subsequent generations of hybrids as associations between loosely linked loci are reduced [76]. If the hybrids are primarily composed of later generation hybrids, then we would not expect to find high estimates of LD. Lastly, we used a Bayesian analysis in Structure v. 2.1 [77] to assign individuals to one of two populations based on recent ancestry. We chose K = 2 and a model of correlated allele frequencies. This analysis was conducted using 100,000 burn-in steps followed by 500,000 steps in the Markov chain from which the posterior probabilities were derived. Individuals were assigned to a single cluster if their probability of membership was greater than 90% and the confidence intervals for inclusion in each cluster were non-overlapping. Otherwise, an individual was considered to be of mixed ancestry.

### Chloroplast Markers

Sequence data were collected at five non-coding chloroplast regions, including four intergenic spacers (*pet*L-*psb*E, *ndh*J-*trn*F, *pet*N-*psb*M, *psb*M-*trn*D), and the intron of *rpS*16. The regions were amplified using universal primers for *pet*L-*psb*E, *ndh*J-*trn*F [78], *pet*N-*psb*M and *psb*M-*trn*D [79] and *rpS*16 [80]. Reaction volumes of 25 or 50 µl included 1 µl of template DNA; 250 µM of dNTP (Promega, Madison, WI, USA); 1× Go-Taq Flexi Buffer (Promega, Madison, WI, USA); 2.5 mM of MgCl_2_; 0.2 µM of each primer (Integrated DNA Technologies Inc., IA, USA); 1.0 µL Bovine Serum Albumin (Promega, Madison, WI, USA) and 2.5 units of Go-Taq Flexi DNA Polymerase (Promega, Madison, WI, USA). The thermal cycler program consisted of 1 cycle of 95°C for 2 min, followed by 35 cycles of 95°C for 1 min, 50°C for 1 min, 65°C for 4 min with a 1°C/8 s ramp from the annealing temperature to the extension temperature [81]. PCR products were verified on 1% agarose gels before cleaning with the Qiaquick PCR cleaning kit (Qiagen, Valencia, CA, USA.).

DNA sequencing reactions were performed with the ABI Prism Big Dye Terminator Cycle Sequencing Ready Reaction kit, v3.1 and subjected to capillary electrophoresis on an Avant-3100 Genetic Analyzer (Applied Biosystems, Foster City, CA, USA) at the University of South Dakota. Cycle sequencing reactions were carried out in 15 µL volumes with Big Dye Terminator v. 3.1 (Applied Biosystems, Foster City, CA, USA) and the PCR primers. Cycle sequencing reactions were purified using precipitation in 3 M NaOH and 95% EtOH. Both forward and reverse directions were sequenced. Sequence chromatograms were edited, and forward and reverse sequences were assembled to produce a single sequence for each sample using Sequencher 4.1 (GeneCodes, Ann Arbor, MI, USA). The data were aligned using Se-al v2.09a [82] with the inclusion of gaps to accommodate insertions/deletions across taxa. Sequences are deposited in GenBank (accessions HM596477-HM596500).

The samples were also genotyped at two single-nucleotide microsatellite regions of the chloroplast genome (i.e., *trn*L intron and *trn*T-*trn*D intergenic spacer region) using primers developed by A. Kuenzi and T. Culley (unpublished data) based on a *trn*L chloroplast sequence for *Schiedea* in the NCBI database (GenBank accession DQ907871) and unpublished sequences for the *trn*T-*trn*D intergenic spacer region (GenBank accession GQ224409; L. Wallace & M. Nepokroeff, unpublished data). The primers were fluorescently tagged and used in multiplexed PCR reactions of all available *S. salicaria*, *S. menziesii*, and hybrid samples using the same conditions as described above for the nuclear microsatellites. The results were then analyzed in Genemarker (SoftGenetics LLC, State College, PA, USA) to identify a single allele for each locus in each sample.

The five sequenced gene regions were concatenated for each individual, and the microsatellite genotypes were also translated into sequence data and included with the concatenated sequences for each individual. A haplotype network was constructed from the fully concatenated data set using statistical parsimony in TCS [83]. Gaps were included as fifth character states. Genetic diversity was determined for each population as well as for *S. menziesii*, *S. salicaria*, and the hybrids using DnaSP v. 5 [84]. We calculated the number of unique haplotypes with and without indels, haplotype diversity, and nucleotide diversity. An AMOVA [73], performed in Arlequin vers. 3.11 [74], was used to test for significant differences in haplotype frequency among *S. menziesii*, *S. salicaria*, and hybrids using pairwise difference as an estimate of genetic distance. Significance of *F*-statistics was determined using 10,000 permutations of the data.

## Supporting Information

Table S1 Number of unique haplotypes and nucleotide diversity observed at each chloroplast locus in each population. For number of haplotypes, the value in parentheses is the number of unique haplotypes when indels are considered in addition to nucleotide substitutions. For nucleotide diversity, standard deviation is reported in parentheses.
(DOC)
