# Supplementary material for: Asymmetrical Gene Flow in a Hybrid Zone of Hawaiian Schiedea (Caryophyllaceae) Species with Contrasting Mating Systems
Source: PLoS One. 2011 Sep 19;6(9):e24845. doi: 10.1371/journal.pone.0024845 (PMC3176226; doi:10.1371/journal.pone.0024845)
Supplement: Table S1 — Number of unique haplotypes and nucleotide diversity observed at each chloroplast locus in each population. For number of haplotypes, the value in parentheses is the number of unique haplotypes when indels are considered in addition to nucleotide substitutions. For nucleotide diversity, standard deviation is reported in parentheses. (DOC) [file pone.0024845.s001.doc]

Table S1. Number of unique haplotypes and nucleotide diversity observed at each chloroplast locus in each population. For number of haplotypes, the value in parentheses is the number of unique haplotypes when indels are considered in addition to nucleotide substitutions. For nucleotide diversity, standard deviation is reported in parentheses.

| Population | ndhJ-trnF | petN-psbM | psbE-petL | psbM-trnD | rps16 | trnD-trnT | trnL | Total |
| --- | --- | --- | --- | --- | --- | --- | --- | --- |
| Number of haplotypes | | | | | | | | |
| 849 | 1 (1) | 2 (2) | 1 (2) | 1 (1) | 1 (1) | 1 (1) | 1 (2) | 2 (3) |
| 949 | 2 (2) | 2 (2) | 1 (1) | 2 (2) | 1 (1) | 1 (1) | 1 (1) | 4 (4) |
| 950 | 2 (2) | 1 (1) | 1 (4) | 2 (3) | 1 (1) | 1 (1) | 1 (2) | 2 (4) |
| *S. menziesii* | 3 (3) | 2 (2) | 1 (4) | 3 (4) | 1 (1) | 1 (1) | 1 (3) | 6 (8) |
| 842 | 1 (1) | 1 (1) | 1 (1) | 1 (2) | 1 (1) | 1 (1) | 1 (1) | 1 (2) |
| 853 | 1 (2) | 1 (1) | 1 (1) | 1 (1) | 1 (1) | 1 (1) | 1 (2) | 1 (3) |
| *S. salicaria* | 1 (3) | 1 (1) | 1 (1) | 1 (3) | 1 (1) | 1 (1) | 1 (2) | 1 (5) |
| Hybrid | 1 (1) | 1 (1) | 1 (1) | 1 (2) | 1 (1) | 1 (1) | 1 (1) | 1 (2) |
| Nucleotide diversity | | | | | | | | |
| 849 | 0 | 0.00094 (0.00011) | 0 | 0 | 0 | 0 | 0 | 0.00013 (0.00002) |
| 949 | 0.00038 (0.00015) | 0.00045 (0.00032) | 0 | 0.00075 (0.00054) | 0 | 0 | 0 | 0.0030 (0.00012) |
| 950 | 0.00088 (0.00034) | 0 | 0 | 0.00146 (0.00056) | 0 | 0 | 0 | 0.00063 (0.00024) |
| *S. menziesii* | 0.0109 (0.00018) | 0.00055 (0.00014) | 0 | 0.00209 (0.00036) | 0 | 0 | 0 | 0.00095 (0.00013) |
| 842 | 0 | 0 | 0 | 0 | 0 | 0 | 0 | 0 |
| 853 | 0 | 0 | 0 | 0 | 0 | 0 | 0 | 0 |
| *S. salicaria* | 0 | 0 | 0 | 0 | 0 | 0 | 0 | 0 |
| Hybrid | 0 | 0 | 0 | 0 | 0 | 0 | 0 | 0 |
